# JLHI dataset: a joint localization and human-to-human interaction dataset for indoor environments with different layouts

**DOI:** 10.1016/j.dib.2026.113106

**Published:** 2026-07-25

**Authors:** Yasir Pulikkal, Yin Hoe Ng, Mohammed Abdulazeem Alameen Abdalla, Muhammad Rizwan

**Affiliations:** Faculty of Artificial Intelligence and Engineering, Multimedia University, 63100 Cyberjaya, Malaysia

**Keywords:** Human-to-human interaction, Indoor localization, Wireless fidelity, Channel state information, Received signal strength indicator

## Abstract

This paper introduces a Wi-Fi based joint localization and human-to-human interaction recognition dataset across diverse indoor layouts, known as the JLHI dataset. It includes 5 distinct interactions performed by 20 different pairs of subjects at 3 locations in 3 different indoor layouts with varying occlusion levels, ranging from open spaces to highly cluttered environments. Each pair of subjects performed 50 trials for each interaction per position per layout, resulting in a total of 45,000 trials (i.e., 20 pairs of subjects x 5 interactions x 50 trials x 3 locations x 3 layouts). To generate and collect the dataset, NI USRP-2901 software-defined radios operating at 5.2 GHz with a sampling rate of 5 MHz were used. The capture Wi-Fi signals contain channel state information with including amplitude and phase across 64 subcarriers, along with received signal strength indicator values, recorded using a custom CSI logger coded using GNU Radio Companion. Unlike existing Wi-Fi based human activity datasets which predominantly focus on single-person activities or human-to-human activities in a fixed layout or position, our dataset bridges this critical gap by providing a multi-user interaction dataset under diverse spatial positions and occlusion conditions. It enables the design of robust machine learning-based models for joint localization and human-to-human activity recognition in dynamic indoor environments that are resilient to environmental changes. Additionally, the dataset offers a realistic benchmark for evaluating model effectiveness across diverse layouts, spatial positions, and occlusion levels, enabling the development of systems that perform reliably in real-world settings.

Specifications Table

**Table:** 

Subject	Engineering
Specific subject area	Wireless sensing and indoor localization
Type of data	Tabular data
How data was acquired	Two NI USRP-2901 units were used, one configured as the transmitter and the other as the receiver, each equipped with two antennas. Data were collected at the receiver during indoor experiments under three indoor spatial layouts. Each layout featured three reference points (RPs), where two participants performed five predefined human-to-human interaction activities at each RP. Layout 1 was unobstructed, Layout 2 positioned three bookshelves placed near the receiver, and Layout 3 placed a large blue board centrally between transmitter and receiver.
Parameters for data collection	Five predefined human-to-human interaction activities were carried out under both line-of-sight (LOS) and non-line-of-sight (NLOS) conditions. For each layout, each pair of participants performed 50 repetitions of every activity at each of the spatial RPs. Both the transmitter and receiver operated at a centre frequency of 5.2 GHz with a 5 MHz sampling rate. From the Wi-Fi packets, channel State Information (CSI), including amplitude in dBm and phase in degrees, and received signal strength indicator were extracted and saved in structured H5 files for post-processing.
Data source location	The experiment was conducted in an indoor lab at the Faculty of Artificial Intelligence and Engineering (FAIE), Multimedia University (MMU), Cyberjaya, Malaysia (2.9264° N, 101.6400° E).
Data accessibility	Repository name: JLHI Dataset Data identification number: 10.17632/xh38tjx3b9.1
Related research article	None

## 1 Value of the Data

• This dataset provides an extensive collection of wireless signal measurements, including both channel state information (CSI) and received signal strength indicator (RSSI), captured from 20 different pairs of participants performing various human-to-human interactions at multiple reference points (RPs) under diverse indoor layouts with varying obstruction conditions. More specifically, it includes five human-to-human activities performed across three indoor layouts, each with three distinct RPs. To the best of the authors’ knowledge, this is the first work to provide a joint localization and human-to-human activity recognition dataset for different layouts of the same indoor area.
• There is relatively limited publicly available dataset for human-to-human interaction recognition dataset. The existing datasets were collected in a single layout and at a fixed point in space. This dataset fills this major gap by providing CSI and RSSI measurements for various human-to-human activities collected in different layouts and positions, introducing spatial diversity and environmental variability.
• The dataset presented in this study supports the development and evaluation of joint localization and human-to-human activity recognition methods that account for indoor layout variation and dynamic indoor conditions.
• The collected dataset serves as a valuable resource for investigating the impacts of indoor layout and position changes on human-to-human interaction recognition systems. Furthermore, it fosters reproducibility and provides a standardized benchmark for comparing emerging methods in this domain.

## 2 Background

This dataset is developed to support the design and evaluation of robust and generalizable models for contactless joint localization and human-to-human activity recognition using Wi-Fi sensing, particularly in complex indoor environments, where vision-based approaches are constrained by occlusions and illumination variations, and are limited by privacy concerns. Carrying out the joint task facilitates a deeper understanding of human behavior and interactions, enabling the development of better intelligent and context-aware systems. Recent studies have demonstrated the feasibility of recognizing daily activities such as walking, sitting, and falling using channel state information (CSI) collected from commodity Wi-Fi devices in conjunction with deep learning models [1,2]. Subsequently, Wi-Fi CSI studies have further emphasized the importance of cross-environment robustness, amplitude-phase feature utilization, hardware-dependent variability, and domain adaptation for practical deployment of device-free sensing systems [3,4]. Publicly available datasets supporting this line of research predominantly focus on single-subject activity execution in controlled indoor environments with single RP and single indoor layout [2,5]. Indoor localization using Wi-Fi sensing has also been extensively studied, where CSI or received signal strength indicator (RSSI) measurements are exploited as fingerprints to estimate user positions at predefined reference points (RPs) [6,7]. In addition to Wi-Fi-only localization, recent work on multi-frequency smartphone positioning and augmentation services has shown that terminal-based positioning performance can be improved through advanced signal processing and correction services [8]. Similarly, multi-sensor indoor positioning studies, including geomagnetic fingerprint distinguishability analysis and robust trajectory matching, suggest that fusing Wi-Fi CSI-RSSI with complementary sensing modalities may further improve localization robustness in complex indoor environments [9].

More recently, attention has shifted toward multi-user activity recognition, where CSI measurements jointly influenced by the movements of multiple individuals are leveraged for prediction, with datasets such as WiMANS [10] facilitating research in this area. However, these datasets primarily characterize parallel individual activities rather than interpersonal interaction and they are collected under fixed position and a single indoor layout. To date, research specifically targeting human-to-human interaction recognition using Wi-Fi sensing remains limited. In [11], Alazrai et al. presented a dataset that captures CSI and RSSI measurements from pairs of participants performing predefined two-person interactions. Nevertheless, the dataset is still confined to a single indoor layout and fixed position. Although joint activity recognition and localization has been explored in [12], it also considers a single indoor layout, thereby restricting their robustness to environmental and spatial variability. To clarify the relationship between the proposed dataset and prior resources, Table 1 summarizes representative Wi-Fi sensing and indoor positioning datasets in terms of sensing modality, number of subjects, activity classes, number of layouts, number of positions, availability of localization labels, and inclusion of CSI amplitude, CSI phase, and RSSI.

**Table 1:** Wi-Fi sensing and indoor positioning datasets.

Ref.	Data Size	Number of Subjects	Number of Activity classes	Environments/layouts	Localization labels	CSI Amplitude/Phase/ RSSI	Primary focus
[1]	NA	NA	8	5 environments	No	Y/Y/N	Location-independent single-user HAR
[2]	3000	30	6	3 environments: 2 LOS and 1 NLOS	No	Y/Y/Y	Single-user HAR under LOS/NLOS conditions
[3]	216	5	6	1	No	Y/Y/N	Amplitude-phase fusion for HAR
[4]	1400	1	7	4 layouts across 3 rooms	No	Y/Y/N	Cross-environment HAR
[5]	12,000	23	9	1 furnished apartment	No	Y/Y/N	Physical-agitation activity recognition
[10]	11,286	6	9	3 environments	Yes	Y/N/N	Multi-user concurrent activity sensing
[11]	3740	6	17	1 furnished room	No	Y/N/N	Dyadic interaction recognition
[12]	1394	1	6	1 indoor environment	Yes	Y/Y/N	Joint gesture recognition and localization
JLHI	45,000	23	5	3 layouts in one physical room	Yes	Y/Y/Y	Joint human-to-human interaction recognition and localization

Existing datasets have contributed substantially to single-user activity recognition, multi-user activity recognition, and indoor localization, whereas datasets specifically designed for human-to-human interaction recognition remain limited. Furthermore, many of these datasets emphasize only one of these aspects or are collected under fixed spatial and layout conditions. In contrast, the JLHI dataset uniquely combines human-to-human interaction labels with reference-point labels across multiple indoor layouts. By providing CSI and RSSI measurements across five predefined interaction types, three RPs, and three indoor layouts with line of sight (LOS) and non-line of sight (NLOS) conditions, the dataset supports controlled evaluation of how spatial position and environmental obstruction affect joint localization and interaction recognition. Therefore, the main contribution of the JLHI dataset is its integrated design for investigating the combined effects of interaction type, subject location, and indoor layout variability in Wi-Fi-based sensing, while acknowledging that broader validation across additional rooms, devices, and participant populations remains necessary.

## 3 Data Description

The dataset is organized into top-level directories named according to participant groups, labeled as Gx, where x represents the group number (e.g., 1, 2, …, 20). Each group contains data recorded from a unique pair of participants performing human-to-human interaction activities. The group structure enables easy indexing and isolation of samples based on subject pairings and experimental conditions. Within each group folder, the data is further categorized into three subfolders named Layout_1, Layout_2, and Layout_3. Each layout corresponds to a distinct spatial configuration and visibility scenario during data collection. These layouts differ in the number and placement of obstructions between the transmitter and receiver, representing varying levels of occlusion in the environment. Each layout folder contains a single h5 file named CSI_all_samples_Gx_Ly.h5, where x corresponds to the group number and y to the layout number. These files include all samples recorded for that specific group-layout pairing, covering all five activities at three spatial RPs. The dataset folder and file structure are presented in Table 2.

**Table 2:** Dataset folder and file structure.

Repository	Description
G1/	Group 1
└——Layout_1/	Data recorded from Group 1 in Layout 1.
└——CSI_all_samples_G1_L1.h5	
└——Layout_2/	Data recorded from Group 1 in Layout 2
└——CSI_all_samples_G1_L2.h5	
└——Layout_3/	Data recorded from Group 1 in Layout 3
└——CSI_all_samples_G1_L3.h5	
G2/	Group 2
└——Layout_1/	Data recorded from Group 2 in Layout 1.
└——CSI_all_samples_G2_L1.h5	
└——Layout_2/	Data recorded from Group 2 in Layout 2.
└——CSI_all_samples_G2_L2.h5	
└——Layout_3/	Data recorded from Group 2 in Layout 3.
└——CSI_all_samples_G2_L3.h5	
…	
G20/	Group 20
└——Layout_1/	Data recorded from Group 20 in Layout 1.
└——CSI_all_samples_G20_L1.h5	
└——Layout_2/	Data recorded from Group 20 in Layout 2.
└——CSI_all_samples_G20_L2.h5	
└——Layout_3/	Data recorded from Group 20 in Layout 3.
└——CSI_all_samples_G20_L3.h5	

Each group-layout file was designed to contain 750 trials, comprising five interactions performed at three RPs with 50 repetitions per interaction-RP combination. Each trial was expected to contain 800 frame records collected over four seconds at 200 frames per second, corresponding to 600,000 rows per file. The complete experimental design therefore comprised 45,000 trials and 36,000,000 expected frame records. The data columns include Coordinates, Action, Frame, FrameType, Sample, Amp_dBm[0–63], Phase_deg[0–63], and RSSI_dBm. An overview of each column, along with its corresponding description, and example value is provided in Table 3. The recorded CSI amplitude and phase readings are raw numerical values produced by the receiver processing pipeline, without additional phase calibration or preprocessing. Each subject appears in at most two groups and is assigned a consistent role to ensure interaction clarity. In every h5 file, the action column uses integer values from 1 to 5 to represent the five predefined interaction activities. The mapping is as follows: 1 for high five, 2 for handshake, 3 for kick with left leg, 4 for punch with the right fist, and 5 for pushing. This standardized numeric representation ensures uniformity across the dataset. The example code and the corresponding supporting documents for utilization of dataset is uploaded to the GitHub repository: https://github.com/MRizHaider/JLHI-Dataset.

**Table 3:** Description of each column in the h5 datasets.

Column	Dimensions	Data Type	Description	Example value
Coordinates	(1,2)	float32	Coordinates of the RP at which the samples were recorded.	(1, 1.5)
Action	Scalar	int8	Activity labels in numerical format, ranging from 1 to 5.	1: high five, 2: handshaking, 3: kicking with left leg, 4: punching with right fist, and 5: Pushing
Frame	scalar	int8	Frame index number within the 4-second recording	1, 2, 3, … , 800
FrameType	scalar	int8	Binary label to distinguish the states, i.e., steady and action state.	0: steady state 1: action
Sample	scalar	int16	Sample number for specific activity.	1, 2, 3, …
Amp_dBm[0–63]	(1,64)	int16	CSI amplitude recorded in dBm from 64 subcarriers.	−49
Phase_deg[0–63]	(1,64)	int16	CSI phase recorded in degree from 64 subcarriers.	−118
RSSI_dBm	scalar	int16	Received Signal strength recorded in dBm.	−53

In addition, the dataset quality was examined for all HDF5 files covering 45,000 trials. All 60 files were successfully readable. The readable files contained 35,360,426 frame records. The examination identified 720,209 missing expected frame positions, corresponding to a record-level loss rate of 2.0006%. No missing scalar values, invalid RP coordinates, invalid action or sample labels, invalid frame indices, or invalid FrameType labels were detected.

Subsequently, from the 45,000 expected trials, 43,998 contained exactly 800 unique frame indices. The 43,954 CSI records from the 43,998 strictly satisfies the completeness criteria, i.e., each of these records contains the 800 unique frame indices without any “Nan” records, corresponding to 97.68% of the intended dataset. CSI amplitude ranged from −200 dBm to 2 dBm, CSI phase ranged from −180° to 180°, and RSSI ranged from −198 dBm to 0 dBm. The mean RSSI was −56.80 ± 6.87 dBm, with a median of −58 dBm and an interquartile range of −58 dBm to −57 dBm. Detailed file- and trial-level quality statistics are provided in the dataset repository. The reported loss rate represents missing stored frame positions and should not be interpreted as direct measurement of over-the-air packet loss.

## 4 Experimental Design, Materials and Methods

### 4.1 Experimental procedure

The data was collected in an indoor laboratory at the Faculty of AI and Engineering (FAIE), Multimedia University (MMU), Cyberjaya, Malaysia. The primary objective is to capture CSI and RSSI for five predefined human-to-human interaction activities. Data acquisition was conducted across three distinct layout configurations that introduced different levels of obstruction between the transmitter and receiver. The JLHI dataset contains only wireless signal measurements, specifically CSI and RSSI values, together with the associated non-identifiable metadata required for data interpretation and analysis. It does not include images, videos, audio recordings, or other identifiable visual recordings of the participants.

**Fig. 1:**
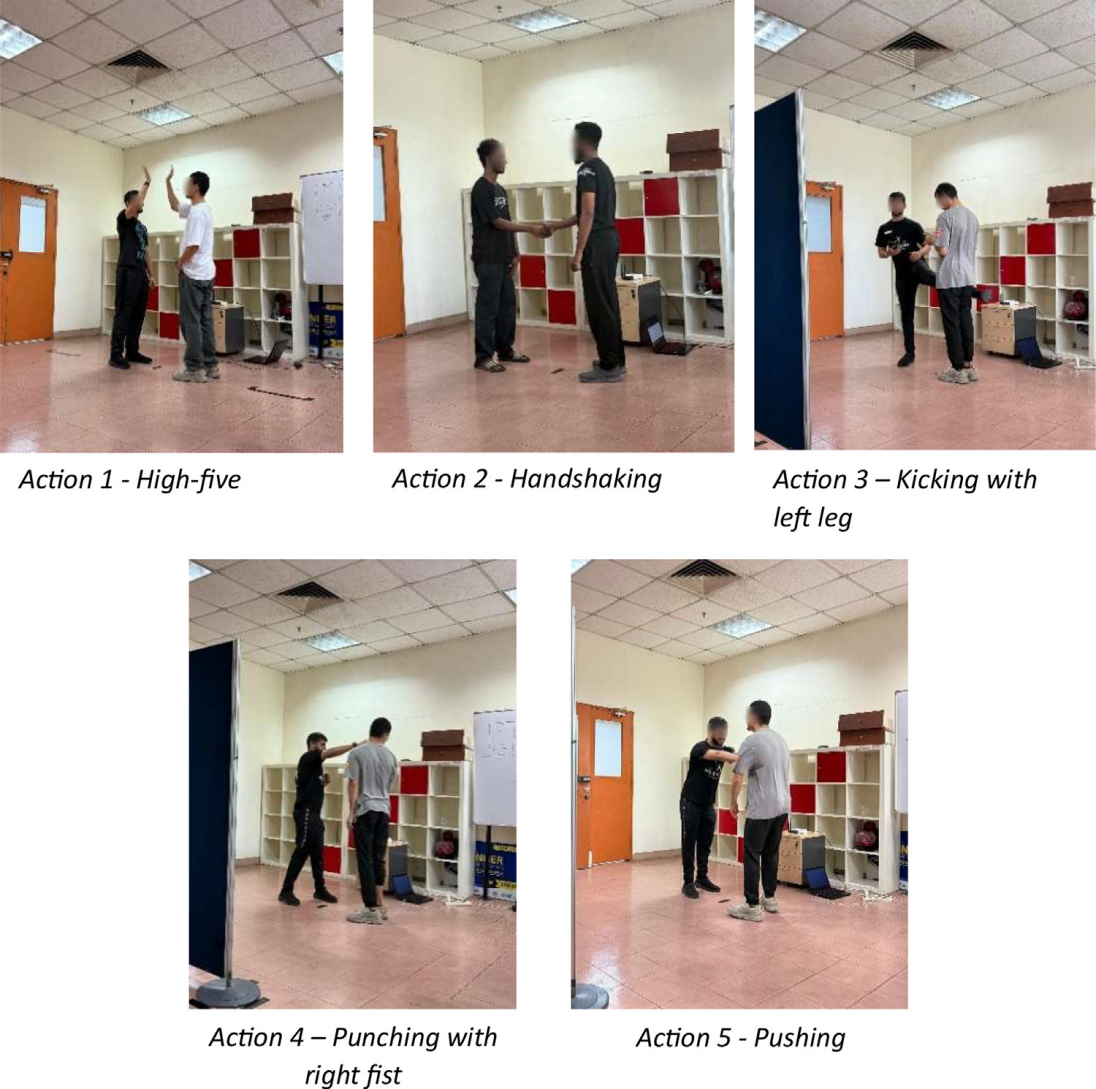
Sample images of the five human-to-human interactions captured in JLHI dataset.

The dataset is structured into groups, where each group corresponds to a unique pair of participants performing five distinct interaction activities, i.e., high five, handshake, kick with the left leg, punch with the right fist, and pushing, as illustrated in the Fig. 1. The images shown in Fig. 1 are provided only for illustrative purposes to demonstrate the predefined interaction classes and are not included in the released dataset. These activities were performed at three spatial RPs in each layout, located at coordinates (1.0, 1.5), (3.5, 1.5), and (2.75, 5.1) measured in meters, relative to an origin defined at the bottom-left corner of the experimental area. The duration of each activity was 4 s, consisting of 1 s of steady state followed 3 s of movement, and the process was repeated 50 times per RP*.*

### 4.2 Equipment and software

**Fig. 2:**
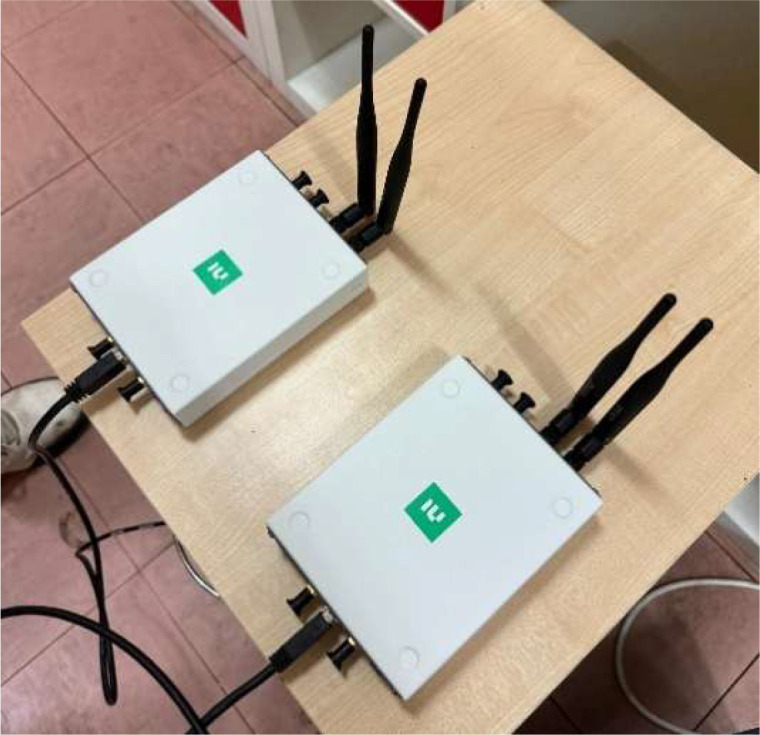
NI USRP-2901 devices employed for transmitting and receiving Wi-Fi packets.

The experimental setup employed two NI USRP-2901 software-defined radio devices, with one configured as the transmitter and the other as receiver, as shown in Fig. 2. Each device was equipped with two omnidirectional antennas and operated at a center frequency of 5.2 GHz with a 5 MHz sampling rate. The 5 MHz value refers to the SDR baseband sampling rate used to digitize the received RF signal for CSI estimation. CSI frames were generated on a per-packet basis, where each CSI frame corresponds to a successfully decoded packet rather than an individual baseband sample. The devices were controlled and configured using GNU Radio Companion (GRC), an open-source graphical software development toolkit for building signal processing flowgraphs. Synchronization was maintained at the acquisition-protocol level by using a fixed packet-generation interval, frame-level sample indexing, and the CSI logger timing signal. No external hardware clock synchronization between the transmitter and receiver was applied [6,7].

The receiver signal processing chain was implemented in GRC using the open-source gr-ieee802–11 module, which provides physical layer decoding capabilities compliant with the IEEE 802.11a/g/p standards. To facilitate CSI extraction, a custom CSI logger was developed in Python to record the CSI amplitude and phase values across 64 subcarriers, together with associated frame-level metadata, including RSSI and sample index. In the experiment, Wi-Fi packets were transmitted at 200 packets per second, with each successfully received packet yielding one CSI measurement across 64 subcarriers. The sample index and frame-level metadata were therefore retained to support frame-by-frame inspection of the recorded CSI sequence and to identify possible missing or irregularly received frames. The CSI data were streamed in real time to the H5 format using fixed-size tagged vectors of length 129, enabling systematic frame-by-frame analysis. During each 4-second acquisition interval, the logger operated at a rate of 200 frames per second [1], yielding 800 rows per sample and 600,000 rows per layout. Specifically, each 4-second sample contains 200 CSI frames/s × 4 s = 800 saved CSI rows. Similarly, for 750 recorded samples per layout per subject, the total number of saved rows is 800 rows/sample × 750 samples = 600,000 rows per layout. The JLHI dataset contains the recorded CSI amplitude and phase values obtained from the receiver pipeline. No additional post-processing, device-to-device phase calibration, or antenna calibration was applied before release. Therefore, users who employ CSI phase information should apply appropriate phase correction or calibration methods according to their own modeling requirements.

**Fig. 3:**
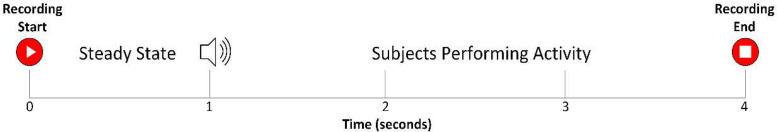
Timing diagram for one activity sample.

**Fig. 4:**
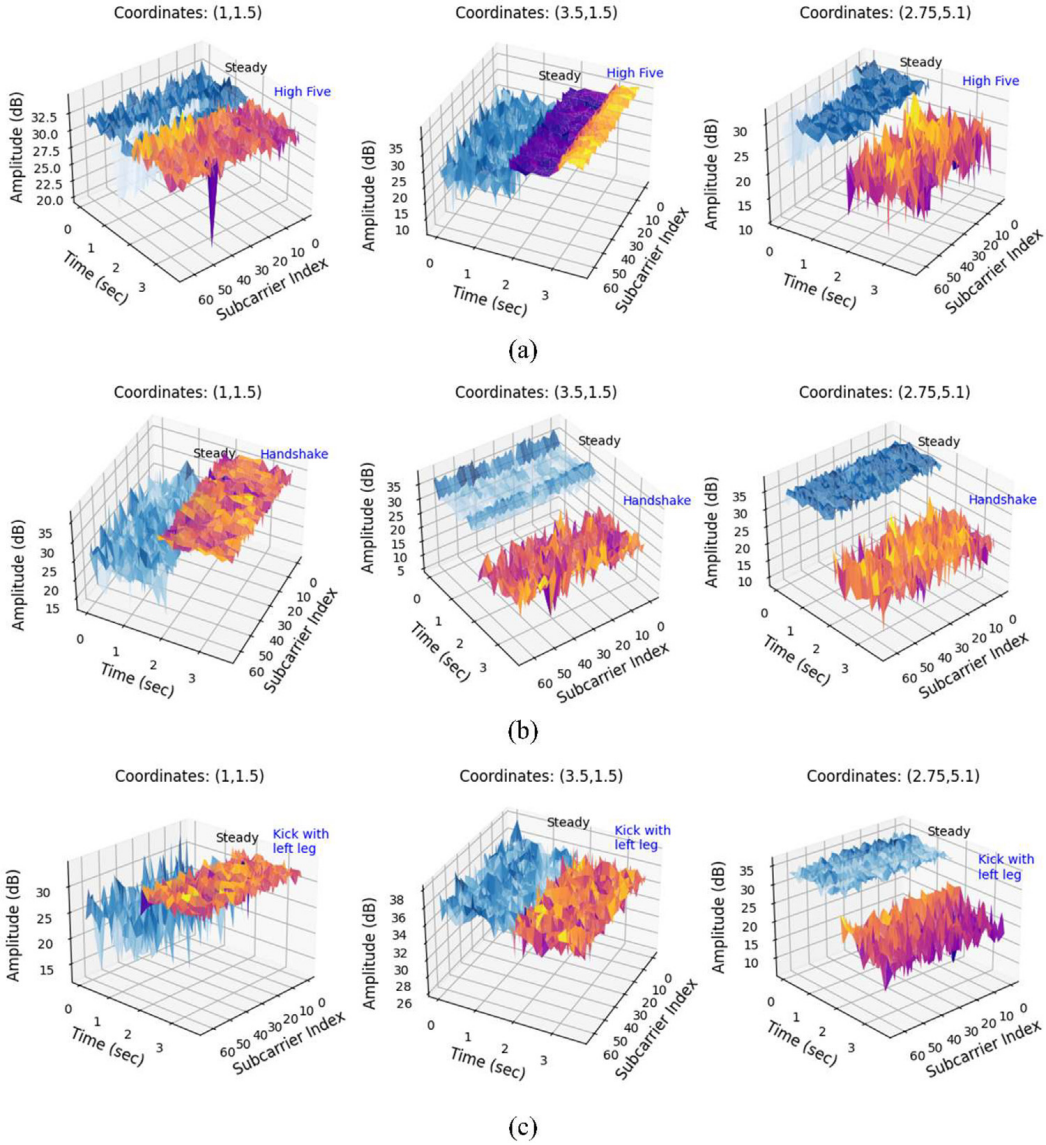

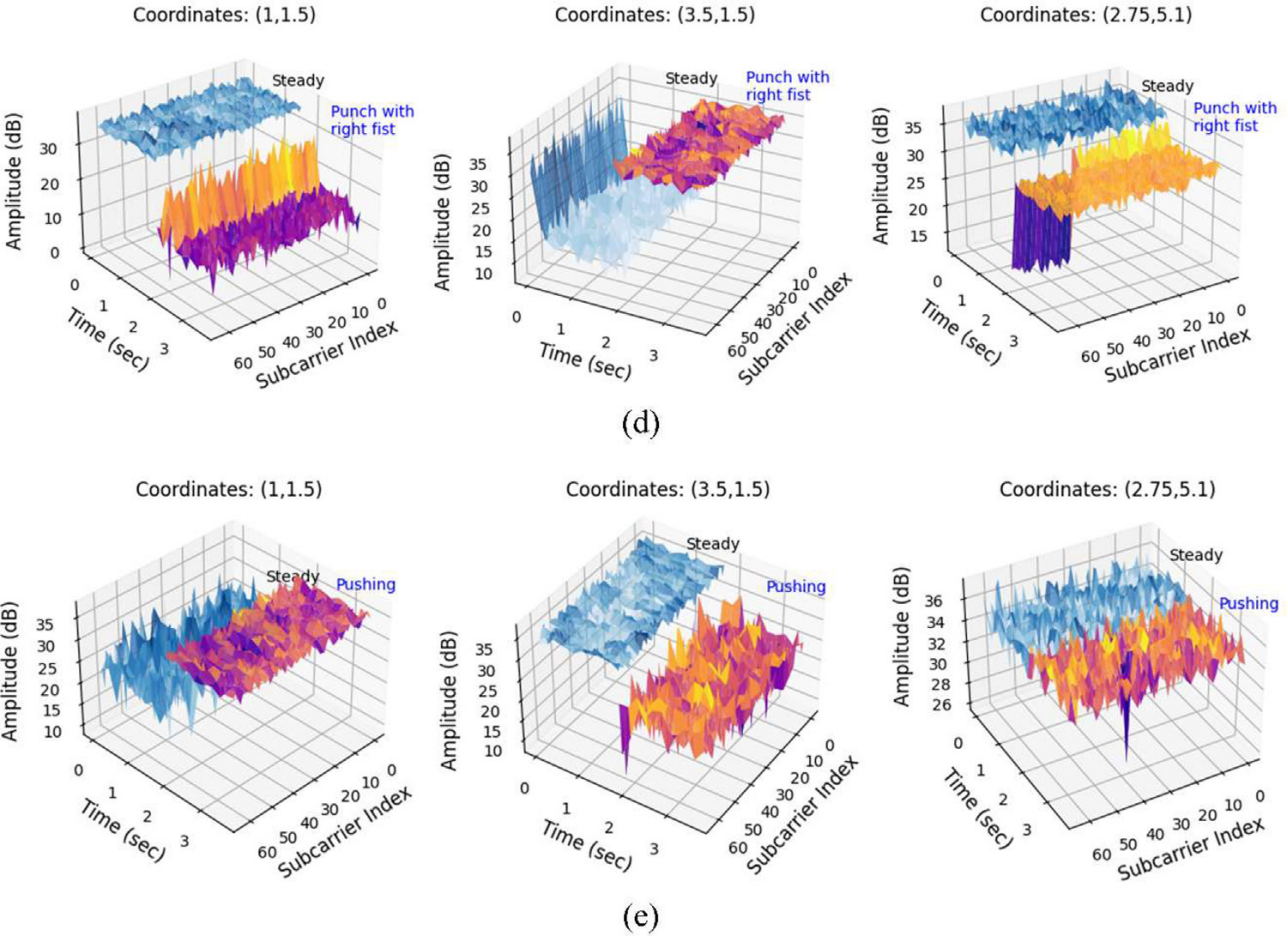
Raw CSI signals for layout 1 at three RPs for (a) high five, (b) handshaking, (c) kicking with left leg, (d) punching with right fist, and (e) pushing.

Furthermore, to ensure precise temporal delineation between the steady and active states of the subjects, an auditory beep was generated by the CSI logger at an exact interval of 1 s to cue the participants to perform the predefined action, as shown in the timing diagram in Fig. 3. Each recording begins with a 1-second steady-state period, during which both participants remain stationary. The beep then signals the onset of the activity, prompting the participants to execute the designated interaction over the subsequent 3 s. Immediately after completing the action, the subjects return to the steady state. For each activity, this procedure is repeated 50 times at every RP and layout, with a 1-second rest interval between consecutive repetitions. This structured acquisition protocol ensures temporal consistency and synchronization across all recorded samples. Fig. 4 illustrates representative raw CSI signals corresponding to five human-to-human interactions collected at three different locations.

### 4.3 Participants and roles

A total of 23 unique participants, identified by subject IDs 1–23, took part in the experiment. To collect the JLHI dataset, 20 interaction groups were formed. In the dataset, “participant” refers to the unique individuals who took part in the study, “subject ID” refers to the anonymized numerical identifiers assigned to those individuals, and “groups” refers to the 20 participant pairs formed for the data collection. Each group consisted of 2 participants, one acting as the active subject who initiates the action and the other as the passive subject who receives or reacts. Subjects were allowed to be either a passive or active subject multiple times, as long as the pairs were different. In case, where two participants appeared together more than once, the activity role between the participants pairs were swapped. For instance, subject IDs 1 and 2 appear once with ID 1 as active and ID 2 as passive, and once with ID 2 as active and ID 1 as passive. This ensures that each group corresponds to unique interaction session, even if the underlying participants pairs are repeated.

The demographic and physical attributes reported in Table 4, namely gender, age, height, and weight, are provided only with anonymized subject IDs to describe the participant characteristics and support transparent interpretation of the dataset. No personally identifying information, such as participant names, contact details, facial images, or institutional identifiers, is associated with these metadata. These attributes are not used to reveal participant identity and should be treated as anonymized descriptive metadata when interpreting or using the dataset. Participants were recruited based on voluntary willingness to take part in the data collection, and gender balance was not a primary design criterion of the study because the main objective was to collect Wi-Fi CSI and RSSI variations associated with human-to-human interaction classes, spatial positions, and layout-dependent obstruction conditions. The CSI and RSSI variations are primarily governed by body motion, relative position, environmental layout and multipath propagation rather than demographic characteristics. Therefore, age and gender are not expected to have a dominant influence on the recorded wireless signal patterns. Table 4 summarizes the participant pairings for each group, their assigned roles, and relevant physical attributes, including the participants’ gender, age, height, and weight.

**Table 4:** Participant pairings within each group, corresponding roles, and associated physical characteristics (gender, age, height, and weight).

Group	Active						Passive			
	ID	Gender	Age	Height	Weight	ID	Gender	Age	Height	Weight
1	1	M	21	174	67	2	M	22	179	67
2	3	M	21	173	69	4	M	22	179	68
3	19	M	24	172	80	20	M	25	173	69
4	7	M	23	181	75	11	M	19	172	72
5	8	M	21	173	66	1	M	21	174	67
6	2	M	22	179	67	7	M	23	181	75
7	9	M	21	178	73	10	M	21	171	60
8	13	M	21	180	70	9	M	21	178	73
9	14	M	19	179	66	15	M	21	170	65
10	4	M	22	179	68	12	F	22	174	55
11	11	M	19	172	72	8	M	21	173	66
12	15	M	21	170	65	3	M	21	173	69
13	16	M	22	179	69	17	M	22	178	64
14	17	M	22	178	64	18	M	20	180	62
15	20	M	25	173	69	16	M	22	179	69
16	21	M	25	172	61	22	M	29	172	52
17	5	F	28	164	58	6	F	27	157	66
18	22	M	29	172	52	2	M	22	179	67
19	2	M	22	179	67	1	M	21	174	67
20	23	M	20	173	106	1	M	21	174	67

### 4.4 Layout configurations and environment

**Fig. 5:**
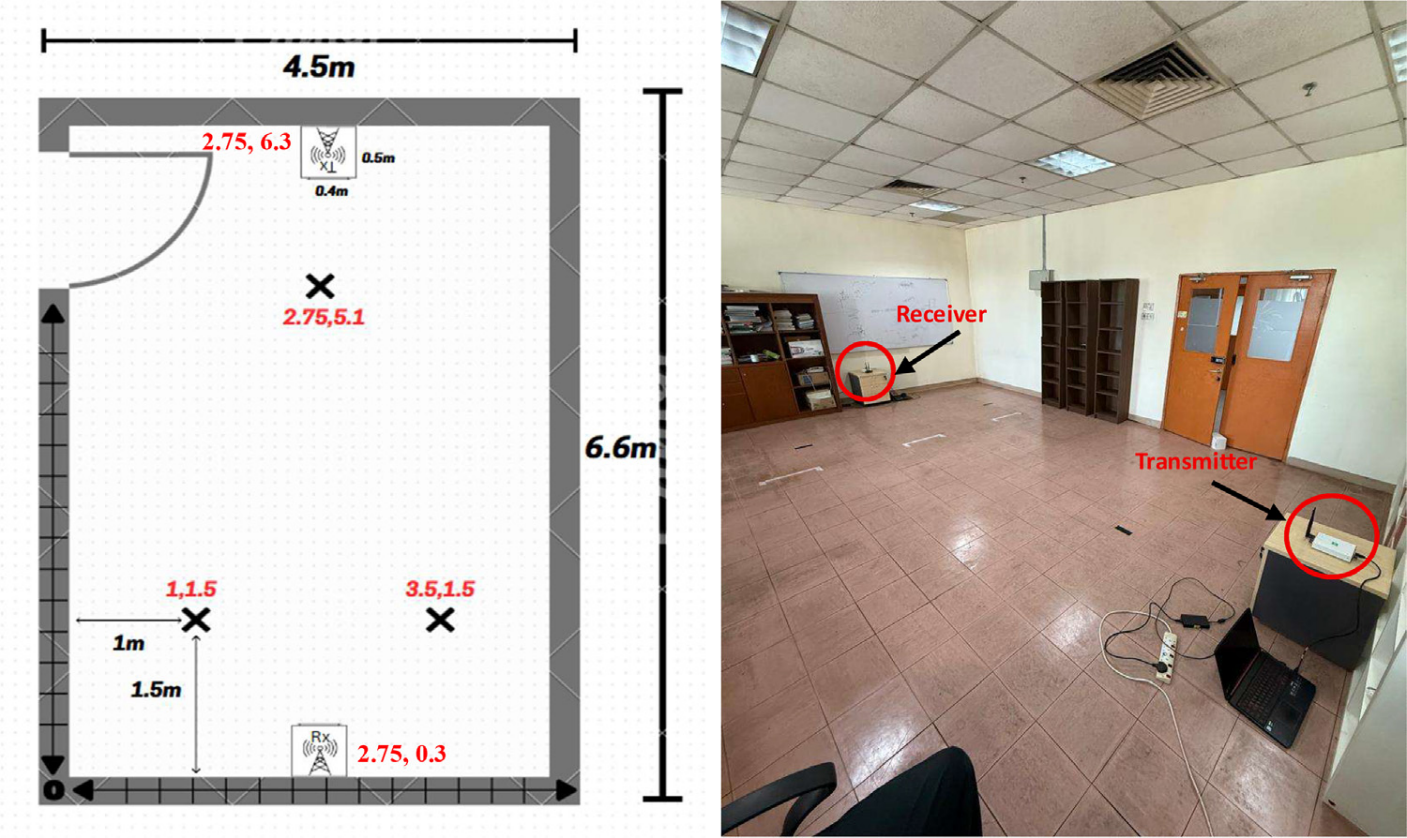
Layout 1.

**Fig. 6:**
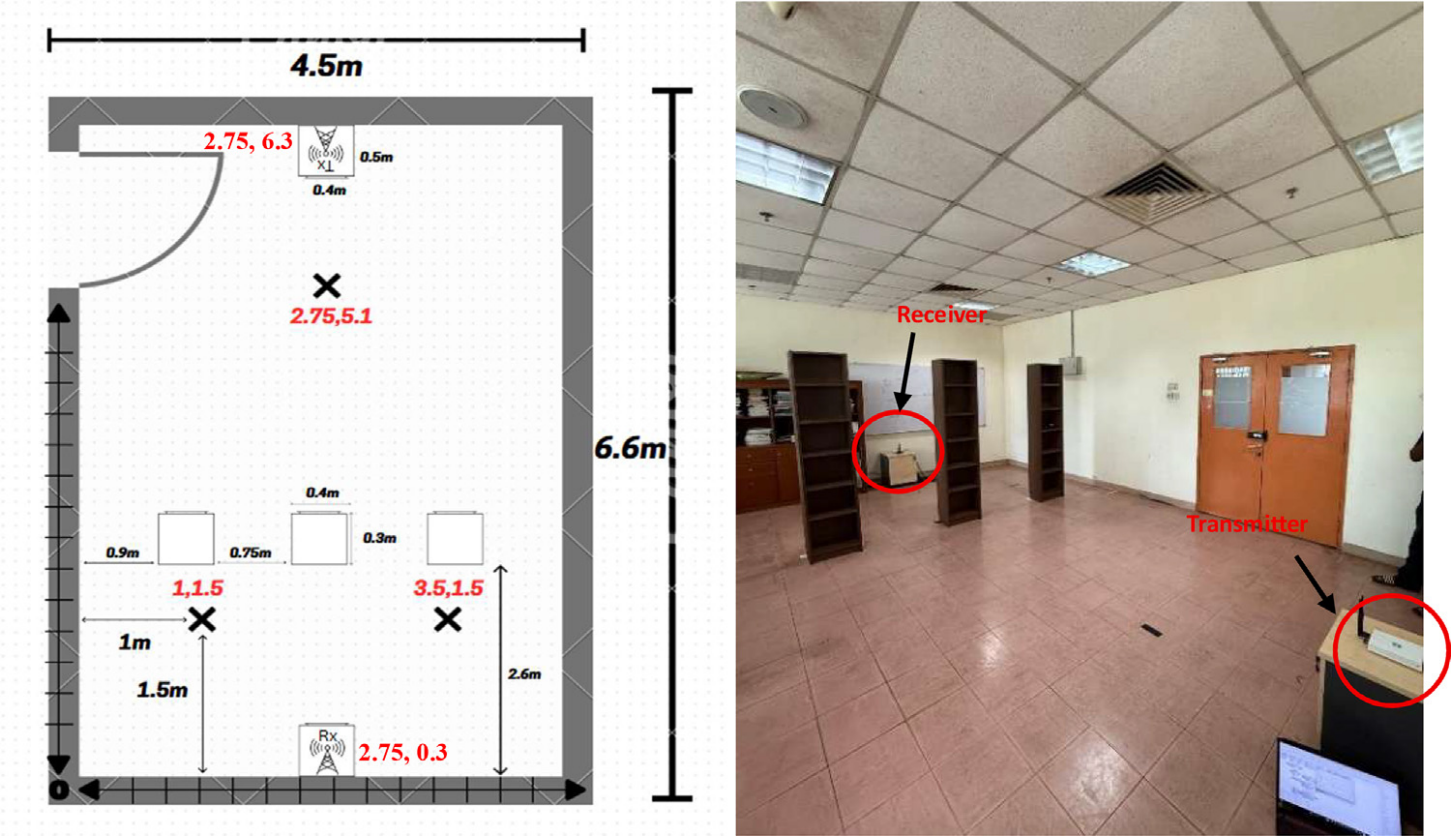
Layout 2.

**Fig. 7:**
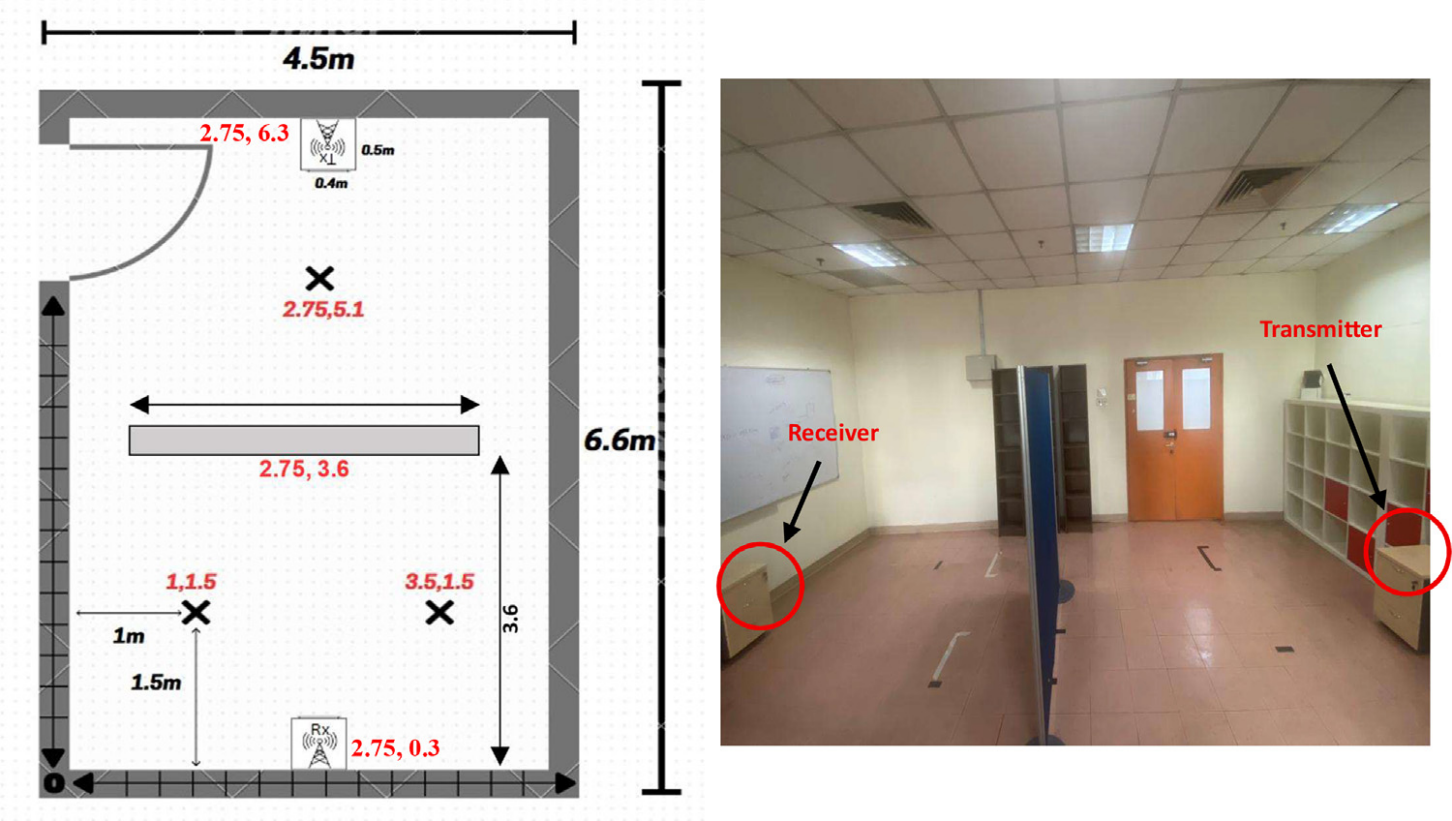
Layout 3.

Data collection was conducted in a laboratory space with dimensions of 6.6 m in length and 4.5 m in width, corresponding to a total area of 29.7 m². To ensure consistency across measurements, all experiments were performed within the same physical environment, and the spatial arrangement of the room was preserved throughout the data acquisition process to minimize environmental variability. As illustrated in Fig. 5, Fig. 6, Fig. 7, the bottom-left corner of the experimental area was defined as the coordinate origin, with all device and RP coordinates expressed in meters. The transmitter (Tx) and receiver (Rx), each equipped with two omnidirectional antennas, were placed at opposite ends of the room at coordinates (2.75, 6.3) and (2.75, 0.3), respectively. Both devices were positioned at an antenna height of 1.2 m above the floor and remained fixed throughout data collection. Participants performed the activities at three predefined spatial RPs in the room for each layout, located at the following coordinates (in meters, relative to the bottom-left corner of the room), which are (1.0, 1.5), (3.5, 1.5), and (2.75, 5.1). At each RP, the two participants faced one another while performing the designated interaction, and their orientation was towards the center of the corresponding RP.

Three indoor layouts were implemented to simulate different levels of obstruction between the transmitter and receiver. Layout 1 featured a clear line-of-sight (LOS) condition with no physical obstruction as shown in Fig. 5. Layout 2 introduced partial obstruction using three evenly spaced bookshelves positioned closer to the Rx side as shown in Fig. 6. The dimensions of each bookshelf were 0.4 × 0.3 × 2.02 m, with their positions relative to the Tx, Rx, and RPs is shown in Fig. 6. Layout 3, illustrated in Fig. 7, established a non-line-of-sight (NLOS) condition using a large vertical panel placed centrally between the transmitter and receiver. The panel had dimesions of 1.5 × 1.82 × 0.0127 m, and its position and orientation relative to the Tx-Rx propagation path are provided in Fig. 7. The obstruction configurations and device positions were kept unchanged during all repetitions conducted within their respective layouts.

## 5 Dataset Evaluation Protocols

To support reproducible benchmarking, the following trial-level evaluation protocols are recommended. It should be noted that frames belonging to the same trial should not be divided between training and testing.

1. **Random trial split:** Complete trials are randomly divided into training and testing sets, preferably using a stratified 80:20 split. This protocol provides a general baseline when training and testing data come from the same overall distribution.
2. **Cross-subject-pair split:** Complete participant groups are held out for testing, while the remaining groups are used for training. This protocol evaluates generalization to unseen interaction pairs.
3. **Cross-layout split:** Data from one layout are used for training and data from another layout are used for testing. This protocol evaluates robustness to changes in obstruction configuration and indoor propagation conditions.
4. **Cross-position split:** Data from selected RPs are used for training, while data from a held-out RP are used for testing. This protocol is mainly suitable for interaction recognition, since a held-out RP would be an unseen class for localization and joint classification.
5. **Leave-one-layout-out:** The model is trained on two layouts and tested on the remaining layout, repeated once for each layout. The mean and standard deviation across the three folds should be reported.

The researchers utilizing the proposed JLHI dataset should report the selected protocol, split configuration, and evaluation metrics. The adoption of these recomended protocols can reduce inconsistent reporting and improve comparability across studies utilizing this dataset.

### 5.1 Dataset validation

To assess the practical usability of the JLHI dataset, baseline experiments were conducted for the joint interaction and localization classification task. In this evaluation, each trial was assigned to one of 15 classes, corresponding to the combination of five human-to-human interaction categories and three reference points. A trial-level random split was adopted, with 35,163 trials used for training and 8791 trials used for testing. To prevent data leakage, all frames belonging to the same trial were assigned exclusively to either the training or testing set.

The H5 files were read directly from the dataset directory structure, and CSI amplitude, CSI phase, RSSI, and label information were extracted for model evaluation. For the classical machine learning models, the CSI measurements were transformed into fixed-length feature representations. Three classifiers were evaluated: k-nearest neighbor (KNN), random forest, and extreme gradient boosting (XGBoost). The KNN pipeline used median imputation, feature standardization, principal component analysis, and distance-weighted five-neighbor classification. The random forest model was configured with 200 estimators, square-root feature selection, and balanced class weights. The XGBoost model was configured with 200 estimators, a maximum tree depth of 6, a learning rate of 0.1, row subsampling of 0.8, column subsampling of 0.8, and a multiclass soft-probability objective.

Table 5 summarizes the dataset validation results. Among the classical machine learning models, random forest achieved the highest accuracy of 71.11%, followed by XGBoost with 70.86% and KNN with 70.57%. These results demonstrate the usability of the JLHI dataset for machine learning evaluation and indicate that the collected CSI and RSSI measurements provide discriminative features for joint human-to-human interaction recognition and RP classification.

**Table 5:** Dataset validation on machine learning models.

Model	Accuracy (%)	Precision	Recall	F1
KNN	70.57	0.7062	0.7058	0.7058
XGBoost	70.86	0.7108	0.7086	0.7090
Random Forest	71.11	0.7139	0.7111	0.7115

## 6 Conclusion

This paper presented the JLHI dataset, a Wi-Fi CSI-based dataset designed for joint localization and human-to-human interaction recognition in indoor environments. The dataset contains CSI amplitude and phase information across 64 subcarriers, together with RSSI values, collected for five predefined human-to-human interactions performed at three reference points under three indoor layout configurations with different occlusion levels. By incorporating both spatial variation and layout-dependent obstruction conditions, the dataset provides a structured benchmark for developing and evaluating machine learning models that jointly infer participant location and interaction type from wireless signals. The JLHI dataset may support future research on device-free indoor sensing applications, including smart environments, assisted living, human–robot interaction, safety monitoring, and context-aware indoor automation systems.

## Limitations

Although the JLHI dataset includes three indoor layouts with different occlusion levels, all recordings were collected within a single physical room, which limits room-to-room and building-level generalizability. The dataset also includes only three reference points per layout and five predefined human-to-human interaction classes, restricting its spatial and interation diversity. In addition, the dataset was collected from a limited participant pool with relatively limited diversity in demographic characteristics, such as age and gender. Future studies involving a broader range of participants and more diverse indoor environments may further enhance the evaluation of Wi-Fi-based joint localization and human-to-human interaction recognition under varying conditions.

## Ethics Statement

This study involved human participants for the collection of wireless signal data during physical interaction tasks. All participants were informed of the study’s purpose and procedures, and written informed consent was obtained prior to participation. The study was conducted in accordance with the Declaration of Helsinki and approved by the Lab Management Committee of the Faculty of Artificial Intelligence and Engineering, under approval number LMC/2025/01 on 21 January 2025.

## Credit Author Statement

**Yasir Pulikkal**: Data curation, Visualization, Formal analysis, Writing – original draft. **Yin Hoe Ng**: Supervision, Project administration, Funding acquisition, Resources, Conceptualization, Methodology, Writing – review & editing. **Mohammed Abdulazeem Alameen**: Data curation, Visualization, Formal analysis, Writing – original draft. **Muhammad Rizwan**: Software, Formal analysis.

## Data Availability

Mendeley DataJLHI Dataset (Original data)
